# Minimum tick size, market quality and costs of trade execution in Vietnam

**DOI:** 10.1371/journal.pone.0285821

**Published:** 2023-05-18

**Authors:** Duc Hong Vo, Bao Doan

**Affiliations:** 1 Research Centre in Business, Economics & Resources, Ho Chi Minh City Open University, Ho Chi Minh City, Vietnam; 2 School of Business & Management, RMIT University, Ho Chi Minh City, Vietnam; Massey University - Albany Campus: Massey University - Auckland Campus, NEW ZEALAND

## Abstract

The Vietnamese government introduced a change in the minimum tick size for stock trading on 12 September 2016 to improve market quality and reduce trade execution costs. The intended effects of this policy have not been widely investigated in an emerging market such as Vietnam. We use data on trade and quote intraday of all stocks listed on the Ho Chi Minh Stock Exchange for the periods before and after the event, with a one-week break from 12/9/2016 to 18/9/2016, for the market to adapt to the new tick size policy. Findings from this paper confirm that the trading cost is reduced following the change to the smallest tick size. However, this is different for large trades executed at the stock price associated with a larger tick size. Furthermore, the findings are robust with a different sample period. These findings imply that introducing a change in tick size in Vietnam in 2016 is desirable for improving market quality. However, the differentiation of these changes in different ranges of stock prices is not necessarily effective for improving market quality and reducing trade execution costs.

## 1. Introduction

The Vietnamese stock market has been considered an important sector of the economy. Indeed, the stock exchange was still operating during the lockdown period in March-April 2020 due to the COVID-19 pandemic in Vietnam. However, to meet the increasing demand of domestic and foreign investors in stock trading in the last ten years, the Ho Chi Minh City Stock Exchange (HSX) and the State Security Commission of Vietnam have put significant effort into improving the market quality. According to [1], market quality can be viewed as the ability of the market to provide liquidity and promote price discovery. In other words, markets with high quality usually have low spreads, high volume, and increased depth.

Among various components for market quality, the minimum tick size is the main component. Therefore, a relationship between the minimum price change and market quality has received much attention from practitioners, policymakers and academics in recent years. The HSX introduced a reduction in tick size on 12 September 2016. However, it is unclear if reducing the minimum tick size leads to a higher level of liquidity, as documented in other markets. Early studies on the decimalization of tick size in the US market in 2001 have reported that a smaller tick size is associated with reducing the bid-ask spread and improving market efficiency. Meanwhile, the tick size and trading volume have been shown to have no relationship between the Taiwan Stock Exchange and Tokyo Stock Exchange. In the context of the HSX, it is crucial to study the impact of minimum tick size on the market quality because the effects may be different for different stocks due to the variation in tick size. In particular, under the current regulations, stocks with prices with a market value of less than 10,000 VND, between 10,000 and 49,950 VND, and above 50,000 VND have tick sizes reduced from 500 VND to 10, 50, and 100 VND, respectively, from 12 September 2016. Suppose the impact of tick size on market liquidity is not uniform for stocks in these three price ranges. In that case, it may suggest that the policies are unsuccessful in promoting liquidity and market quality in the Vietnamese context. We expect that the effect on market liquidity from a reduction in tick size is most substantial for the smallest tick size of 10 VND and decreases with the larger tick size. Intuitively, the tick size increases lead to larger equity bid-ask spreads, reducing liquidity. This study examines the effect of decimalization on trade execution cost and market quality in Vietnam.

Following this introduction, section 2 discusses relevant empirical studies on the issue for other countries. Research methodology and data are then presented in section 3. Finally, section 4 discusses the empirical findings from our analysis, followed by the conclusions and policy implications in section 5 of the paper.

## 2. Literature review

### 2.1. Trade execution costs and change in tick size

Various studies have been conducted to examine the relationship between changes in tick size and trade execution costs. For example, theoretically, Harris [2] and Foucault, Kadan, and Kandel [3] consider that an optimal tick size may exist, and it must be small enough to diminish trade execution costs accompanied by bid-ask spreads. In addition, an optimal tick size should not be equal to zero because it encourages investors to provide more liquidity into the market. Besides, theoretical predictions indicate a convex relationship between tick size and relative bid-ask spreads.

Based on empirical evidence, findings from many papers report a significant and positive relationship between changes in tick size and transaction costs. For example, Rindi and Werner [4] report that an increase in tick size leads to larger bid-ask spreads of traded stocks. The study uses the difference-in-difference technique with panel data from the US-listed stocks around the introduction of the US tick size pilot in 2016. Griffith and Roseman [5] show that the wider tick size can increase transaction costs in the limit order book on the Nasdaq exchange using the same methods. However, a few studies have been conducted on developing countries. For example, Ke, Jiang, and Huang [6] examine the impact of the larger tick size on intraday stock price behaviour for securities listed on the Taiwan Stock Exchange throughout 1998–1999. Their findings suggest that bid-ask spreads increase when the tick size increases.

A reduction in tick size should bring the opposite impacts of a tick size increase. A tick size reduction has lower transaction costs as spreads narrow in the US market context [7]. This finding is supported by [8]. They argue that a reduction in transaction costs is accompanied by a switch from limit orders to market orders among traders in order-driven markets. In Asian countries, Ahn et al. [9] argue that both quoted and effective spreads decline significantly after introducing the tick size reduction on Tokyo Stock Exchange.

The positive relationship between tick size and transaction costs can be explained by the tick size restricting the price traders can order. As a result, price competition among traders is limited, and traders hardly achieve the best bid/offer price in the case of only one tick of the spread. A tick size reduction leads to tighter bid-ask spreads [10]. Besides, many market orders are dominated by small traders who frequently trade at the best-quoted price, and smaller spreads will minimize their transaction costs. Harris [10] concludes that the bid-ask spread reduction eliminates the binding constraint on spreads.

Another possible explanation documented in many studies is that liquidity providers can obtain a monopoly in economic rents from price discreteness. As such, abnormal profits can be made as long as these providers provide more liquidity to the market. However, when the tick size is reduced, liquidity suppliers are encouraged to trade with limit orders, reducing the liquidity cost component within the bid-ask spreads. In other words, the reduction of the bid-ask spreads comes from declining economic rents [11, 12]. This theory seems consistent with the view raised in other studies, such as [13, 14], that the competition between liquidity providers and liquidity demanders probably reduces bid-ask spreads.

### 2.2. Market quality and changes in tick size

Market quality can be defined as the market’s ability to provide liquidity and promote price discovery [1]. Hence, markets with high quality usually have some characteristics such as low spreads, high volume and increased depths. Besides, the minimum tick size is considered an essential component of market microstructure that potentially affects market quality. For that reason, a relationship between a minimum price change and market quality has received much attention from scholars in recent years.

Theoretically, Harris [15] argues that the smaller tick size leads to a reduction in liquidity. For stocks with a tick-constrained spread, the bid-ask spread equals one tick with high quoted depth at the best prices since specialists and traders find liquidity provision profitable. In this context, time priority is key to execution. Harris [15] also suggests that a reduced tick size will narrow the quoted spreads and quoted depth on the constrained stocks due to reducing the marginal profitability of supplying liquidity. Harris further assumes the tick size as the subsidy paid to liquidity supplied. As such, a decline in the subsidy will lead to an alteration in the level of liquidity supplied. In particular, liquidity suppliers are willing to reduce the bulk of shares they order at any given price and alter their shares to limit prices to retake some lost profits. In addition, traders can step ahead of the existing quotes and limit orders to obtain better places in the queue for trading [16, 17]. A recent study by Pham et al. [18] investigates the effect of introducing the VN30 futures contract on the liquidity of thirty blue-chip underlying stocks in the Vietnamese stock market. Their findings indicate that the quoted spread and the Amihud illiquidity of the VN30’s component stocks increased after introducing index future trading in the Vietnamese stock market.

Several empirical studies on tick size reductions from the international markets have examined and verified a prediction from Harris’s [15] study. For example, based on the event-study technique, Bessembinder [19]; Chung, Charoenwong, and Ding [20] report that a smaller tick size reduces the bid-ask spread and improves price efficiency. In contrast, Bacidore, Battalio, and Jennings [21] suggest that the smaller limit orders harm liquidity in the US context. Pavabutr and Prangwattananon [22] examine the effect of a tick size reduction on bid-ask spreads, depths, and trading volume in Thailand after the Thai government introduced a tick size reduction on stocks priced below THB 25. Their findings confirm that the tick reduction produces similar empirical results found in markets where institutional investors are more dominant. Tick reduction on the Thai stock market is associated with reduced spreads, quoted, and accumulated market depths. Besides, to differentiate the impact of a tick size reduction on illiquid and liquid stocks, Buti et al. [23] predict that market quality and welfare had deteriorated for illiquid stocks but improved for liquid stocks based on the European and US data. Besides, in Asian countries, Ke, Jiang, and Huang [6] suggest that a relationship between the tick size and trading volume is insignificant using a sample of listed stocks on the Taiwan Stock Exchange. This finding is supported by [9] using the Tokyo Stock Exchange data.

Extant literature has provided ample empirical evidence of the impact of tick size increases on enhancing market quality. Goettler, Christine and Uday [8] argue that an increase in average tick size will result in an increased depth, a reduction in volume and a widening of spreads. In contrast, Duane [24] suggests that an increase in average tick size is associated with increased trade size. In addition, Anshuman and Kalay [11] and Kadan [25] confirm that a larger tick size will promote liquidity provision and accelerate price discovery. Pham [26] investigates the changes in liquidity in Vietnam following a tick size reduction. The findings confirm that market liquidity in Vietnam significantly decreased in the post-event of the tick size reduction on 12 September 2016. However, the market liquidity is not enhanced in the Vietnamese stock market. The tick size reduction positively affects liquidity related to the tightness dimensions but not the depth dimensions.

In summary, various empirical studies have been conducted to provide empirical evidence on the relationship between tick size with trade execution costs and market quality. However, the focus of those studies is on the developed markets. Emerging markets such as Vietnam have been mainly ignored in empirical analysis on this important issue. As such, this study contributes to the existing literature on this vital issue for emerging markets because our study uses a sample including listed stocks in Vietnam’s stock market.

## 3. Research method and data

### 3.1. Method

The paper considers two procedures, namely equal- and volume-weighted approaches, to calculate the main interest variables. First, each trade in the stock is weighted equally or by the number of shares transacted to compute the daily mean for each stock. Second, the cross-sectional daily mean is calculated by the equal- or volume-weighted average to obtain time series market data. We note that the Vietnamese stock market has two stock exchanges: Ho Chi Minh Stock Exchange (HSX) and Hanoi Stock Exchange (HNX). While the stocks traded on HSX were affected by the tick size rule changes, those traded on HNX were not. Therefore, this provides an excellent opportunity to estimate the impact of the policy change when controlling for other macroeconomic conditions. from using the Difference-in-Difference (DID) approach. That is, for a given variable, we run the following regression:

$$y_{t}=\;\alpha +\beta_{1}event_{t}+\beta_{2}HSX_{t}+\beta_{3}event_{t}\times \;HSX_{t}+\epsilon_{t}$$

where *event*_*t*_ equals 1 in days following the event and 0 otherwise, *HSX*_*t*_ equals 1 for stocks listed on the HSX and 0 otherwise. The coefficient *β*_3_ illustrates the impact of minimum tick size change on the dependent variable *y*_*t*_ with the DID approach. Therefore, our results section will mainly focus on the coefficient *β*_3_. Our approach to evaluating statistical significance differs from [19] in the second step. In particular, for each stock, the daily time-series mean is calculated by the equal- or volume-weighted average from daily values so that we have the cross-sectional data across stocks before and after the policy change. This is because the new regulation on the tick size change in HSX requires that stocks with prices less than VND 10,000 (< VND 10,000), between VND 10,000 and VND 49,950 (between VND 10,000 and VND 50,000), and above VND 50,000 (> VND 50,000) have a tick size of VND 10, 50, and 100, respectively, from 12/9/2016.

In the pre-event period, the tick size of VND 500 was applied to all stock prices. In other words, the minimum tick size applied for a given stock will change daily, depending on the stock price on a given day. We show later that this policy change does not improve the spread in a price range between VND 10,000 and VND 50,000. In contrast, the minimum tick size change in the US stock market is uniformly applied to all stock prices, making the cross-sectional data across stocks over the pre-and post-event periods more relevant. If this were applied to our context, one would calculate the daily average stock price to allocate the stock to the price basket of < VND 10,000, between VND 10,000 and VND 49,950, and > VND 50,000 over the pre-and post-event period. Therefore, it removes the daily variation in minimum tick size change on that stock, especially for those whose daily price fluctuates from one price basket to another.

However, as a robustness check, we control for the cross-sectional determinants of stock liquidity in Stoll [27] in the DID regression above, namely the (natural) log of the average daily dollar volume, the daily return variance over the prior 12 months, the log of the market value, log of closing price, log of the average number of trades per day, and average daily percentage imbalance between the buy- and sell-volume. For each determinant, we first take the daily values in a given stock and calculate the simple average across stocks to represent the market’s daily value.

### 3.2. Data

We collect the trade and quote intraday data of all stocks traded on HSX and HNX from Datascope Refinitiv from 15/7/2016 to 11/11/2016, whose continuous trading hours range from 9:15 to 11:30 (9:00 to 11:30) and 13:00 to 14:30 (13:00 to 14:30 in the HSX (HNX) and the currency is Vietnam Dong (VND). We retain the positive bid/ask quotes and positive bid-ask spread as valid quotes. For trade data, if all trades co-occur and price, we aggregate all trading volumes into one. We follow [28] to infer the trade direction (buy or sell trades). If the month-end stock price over the sample period is less than VND 3,000 or greater than VND 300,000, we drop that stock out of the analysis. The daily value of other determinants is obtained from Datastream Refinitiv over the same sample period. To evaluate the effect of the tick size change effective on 12/9/2016, we studied the 40 trading days (8 weeks) before and after this event, 15/7/2016 to 11/9/2016 and 19/9/2016 to 11/11/2016, respectively. We leave out one week from 12/9/2016 to 18/9/2016 for the market to adapt to the new tick size policy.

## 4. Empirical results

### 4.1. Quoted bid-ask spread

Table 1 reports the full sample results and different price ranges. The first panel shows that the quoted spreads narrow after the tick size change with equal- and volume-weighted approaches at a 1% significance level. This finding is consistent with Bessembinder’s [19] findings and references therein. An exception is that the equal-weighted result in the price range of VND 10,000–50,000 is negative but not statistically significant. Overall, the quoted spread decreases by VND 59 in the equal-weighted approach and substantially by VND 76 in the volume-weighted method, and the largest decrease of VND 464 and VND 622 in the two approaches takes place on stock prices above VND 50,000.

**Table 1:** Average quoted bid-ask spreads and share prices.

	Equal-weighted				Volume-weighted			
	Constant	Event_t	HSX_t	Event_t *HSX_t	Constant	Event_t	HSX_t	Event_t* HSX_t
	Quoted spread in VND							
All stocks	567***	24*	-177***	-59***	158***	-1	2	-87***
	[66.15]	[1.7]	[-17.3]	[-3.57]	[29.19]	[-0.09]	[0.34]	[-10.8]
Price above VND 50k	1538***	282***	-602***	-464***	607***	213***	81***	-622***
	[22.45]	[2.86]	[-8.57]	[-4.52]	[22.22]	[3.44]	[2.83]	[-9.74]
Price between VND 10k and VND 50k	578***	28*	-247***	-26	152***	1	-22***	-44***
	[66.12]	[1.73]	[-23.17]	[-1.44]	[23.52]	[0.11]	[-3.08]	[-4.48]
Price less than VND 10k	251***	-10*	-115***	-20***	125***	1	-25***	-79***
	[66.12]	[-1.79]	[-26.6]	[-2.96]	[32.73]	[0.06]	[-6.32]	[-8.97]
	Average share price							
All stocks	21535***	618***	8352***	-27	16345***	-334	7055***	-377
	[224.66]	[4.45]	[60.64]	[-0.14]	[66.58]	[-0.8]	[12.43]	[-0.44]
Price above VND 50k	81237***	8471***	3749***	-6650***	79377***	15120***	15290***	-3347
	[138.2]	[8.17]	[5.33]	[-5.86]	[58.3]	[7.08]	[7.56]	[-1.08]
Price between VND 10k and VND 50k	20400***	827***	3101***	-609***	17037***	969***	8180***	-184
	[218]	[6.92]	[28.69]	[-4.37]	[108.75]	[3.27]	[28.13]	[-0.35]
Price less than VND 10k	6905***	-229***	-99***	-30	6792***	-205	-649***	-365**
	[344.35]	[-7.78]	[-2.71]	[-0.6]	[95.68]	[-1.23]	[-7.22]	[-2.01]
	Quoted spread in % of the price							
All stocks	3***	0.06	-1.44***	-0.22***	1.15***	0.15**	-0.13***	-0.79***
	[108.22]	[1.22]	[-44.56]	[-3.79]	[36.5]	[2.24]	[-3.79]	[-11.26]
Price above VND 50k	2.15***	0.18	-0.99***	-0.42***	0.79***	0.2*	-0.01	-0.68***
	[20.39]	[1.28]	[-9.24]	[-2.86]	[25.25]	[1.94]	[-0.4]	[-6.39]
Price between VND 10k and VND 50k	2.77***	0.05	-1.3***	-0.04	0.91***	-0.02	-0.32***	-0.18***
	[65.27]	[0.74]	[-26.94]	[-0.54]	[30.62]	[-0.54]	[-9.83]	[-4.1]
Price less than VND 10k	3.72***	-0.01	-1.59***	-0.54***	1.97***	0.17	-0.24***	-1.52***
	[80.3]	[-0.13]	[-29.42]	[-5.95]	[35.01]	[1.1]	[-4.1]	[-9.9]

The table presents the coefficient estimates and t-statistics in square brackets of the following regression:

*y*_*t*_ = *α* + *β*_1_*event*_*t*_ + *β*_2_*HSX*_*t*_ + *β*_3_*event*_*t*_ × *HSX*_*t*_ + *ϵ*_*t*_, where *event*_*t*_ equals 1 in days following the event and 0 otherwise, *HSX*_*t*_ equals 1 for stocks listed on the HSX and 0 otherwise, and the coefficient *β*_3_ illustrates the impact of minimum tick size change on the dependent variable *y*_*t*_ with the DID approach. The daily average of quoted spreads is measured for each stock on a time-weighted basis. The quoted spread and close price are averaged across stocks daily by equal- and volume-weighted approaches. Before and after the tick size change, the period covers eight weeks before 12/9/2016 and eight weeks from 19/9/2016, respectively. Standard errors are adjusted for heteroskedasticity.

***, **, and * denote the statistical significance at the 1%, 5%, and 10% level, respectively.

The second panel displays the average transaction prices that do not change much over the sample period. While the stock prices above VND 10,000 decrease by VND 609 or VND 6,650 at a 1% significance level in the post-event period on an equal-weighted basis, the stock prices less than VND 10,000 decrease by VND 365 at a 5% significance level. Considering that investors are more concerned with trading costs as a percentage of share prices than with the absolute amount per share, the last panel reports the percentage quote spread calculated as the quoted spread relative to the trade price. We find the decreasing patterns of percentage quoted spreads as in the case of quoted spreads at the 1% significance level, except for the equal-weighted value in the price range between VND 10,000 VND and VND 50,000. On average, the percentage spread decreases by 0.22% on an equal-weighted basis and from 0.79% on a volume-weighted basis. The substantial decrease in the volume-weighted value is also documented for different price ranges: 1.52%, 0.18%, and 0.68% for prices less than VND 10,000, between VND 10,000 and VND 50,000, and above VND 50,000. This finding is consistent with the view that introducing tick size change designs reduces trading costs, as measured by the quoted spreads.

### 4.2. Executions inside and outside the quotations

Suppose trades are often executed at prices lower than the quotes, especially when institutional traders can negotiate trade prices in the HSX. In that case, the quoted bid-ask spread is not a proper measure of trade execution cost. To investigate this, we document the percentage of executions at prices outside the contemporaneous quotations in Table 2. Aligned with [19], more trades are executed outside the quotes after the tick size change, regardless of different price ranges.

**Table 2:** Trade prices relative to best quotes.

	Equal-weighted				Volume-weighted			
	Constant	Event_t	HSX_t	Event_t* HSX_t	Constant	Event_t	HSX_t	Event_t* HSX_t
	% of outside the quotes							
All stocks	6.7***	0.73**	-1.48***	2.69***	3.22***	0.44	-1.27***	3.92***
	[37.07]	[2.53]	[-7.11]	[7.85]	[8.36]	[1.01]	[-3.19]	[7.04]
Price above VND 50k	9.88***	0.53	-6.15***	4.97***	9.35***	0.98*	-8.16***	5.33***
	[17.85]	[0.61]	[-10.39]	[5.43]	[24.58]	[1.65]	[-21.03]	[8.18]
Price between VND 10k and VND 50k	7.15***	0.49	-0.56**	0.86**	3.11***	0.14	-0.29	2.63***
	[29.17]	[1.37]	[-2.01]	[2.02]	[6.58]	[0.29]	[-0.59]	[3.76]
Price less than VND 10k	4.87***	1.39***	-2.25***	6.02***	2.59***	1.43***	-1.86***	4.92***
	[15]	[2.6]	[-5.74]	[9.19]	[11.4]	[3.11]	[-7.73]	[7.47]
	% of small trades outside the quotes							
All stocks	58.72***	1.35	-48.96***	-7.36***	37.45***	-0.34	-19***	-7.26***
	[77.33]	[1.15]	[-55.92]	[-5.85]	[31.07]	[-0.18]	[-11.32]	[-3.21]
Price above VND 50k	54.26***	0.19	-46.04***	-5.91**	48.5***	-8.86***	-31.53***	0.14
	[28.94]	[0.07]	[-22.4]	[-1.96]	[26.72]	[-3.13]	[-10.36]	[0.04]
Price between VND 10k and VND 50k	58.11***	0.05	-49.01***	-5.06***	40.31***	0.26	-19.45***	-9.91***
	[59.33]	[0.04]	[-45.79]	[-3.34]	[23.79]	[0.11]	[-8.78]	[-3.42]
Price less than VND 10k	65.14***	2.2	-50.13***	-13.37***	26.86***	2.83	-12.98***	-6.93*
	[29.38]	[0.79]	[-19.11]	[-4.26]	[12.48]	[0.96]	[-4.38]	[-1.84]

The table presents the coefficient estimates and t-statistics in square brackets of the following regression:

*y*_*t*_ = *α* + *β*_1_*event*_*t*_ + *β*_2_*HSX*_*t*_ + *β*_3_*event*_*t*_ × *HSX*_*t*_ + *ϵ*_*t*_, where *event*_*t*_ equals 1 in days following the event and 0 otherwise, *HSX*_*t*_ equals 1 for stocks listed on the HSX and 0 otherwise, and the coefficient *β*_3_ illustrates the impact of minimum tick size change on the dependent variable *y*_*t*_ with the DID approach. The daily percentages of trades in each stock completed outside the quotes are computed and averaged across stocks. The daily percentages of shares transacted in each stock at prices outside the quotes are computed and averaged by a volume-weighted approach across stocks. Small trades are those with a size less than or equal to the most recent quote on the same side of the market. Before and after the tick size change, the period covers eight weeks before 12/9/2016 and eight weeks from 19/9/2016, respectively. Standard errors are adjusted for heteroskedasticity.

***, **, and * denote the statistical significance at the 1%, 5%, and 10% level, respectively.

On average, the percentage of such trade executions increases dramatically by 2.69% on an equal-weighted basis and 3.92% on a volume-weighted basis at a 1% significance level. Among the three-price range category, the price range between VND 10,000 and VND 50,000 displays the smallest increase in such trades, i.e. 0.86% and 2.63% on the equal- and volume-weighted basis, respectively, and the results are statistically significant at 5% level. The results indicate the declining relevance of quotations for trade execution costs after the tick size change.

The second panel in Table 2 reports the results on trades outside the quotes with a size less than or equal to the corresponding quote size. In general, we find that outside the quote, executions that are smaller than quote sizes occur less often than before the tick size change. Most results are statistically significant at a 1% level, except for the price range above VND 50,000 in the volume-weighted method. This finding implies that the overall increase in trades outside the quotes is not attributable to quote size. Bessembinder [19] notes that the apparent increase outside the quote executions can reflect rapid trading and quote updates, resulting in difficulties matching trade prices and quotes prevailing at the trade execution time. In the context of HSX stocks, if trades outside the quotes are typically negotiated between institutional investors days ahead of the actual trade, especially for stocks with limited foreign ownership or owned by institutional investors, this can make the issue above less relevant.

### 4.3. Effective bid-ask spreads

The previous section documents that the percentage of trades completed outside the quotes has increased since the tick size reduction, implying that changes in trading cost can be different from that implied by changes in quoted spreads. To better reflect the trading cost, we employ the effective bid-ask spread, defined for each trade as twice the absolute difference between the trade price and the mid-point of the contemporaneous bid and ask quotes, and report the results in Table 3. The effective spread decreases by VND 62 (0.22%) on an equal-weighted basis at a 1% significance level. However, the result becomes less statistically significant on a volume-weighted basis. To explain the weak statistically significant change in the volume-weighted effective spread, we note that the increase in trades outside the quote combined with the decrease in share price can offset the striking decrease in quoted spread completely, leaving the percentage effective spread unchanged.

**Table 3:** The average effective bid-ask spreads.

	Equal-weighted				Volume-weighted			
	Constant	Event_t	HSX_t	Event_t* HSX_t	Constant	Event_t	HSX_t	Event_t* HSX_t
	Effective spread in VND							
All stocks	631***	28*	-179***	-62***	259***	137**	-21	-68
	[65.31]	[1.83]	[-15.33]	[-3.34]	[10.45]	[2.33]	[-0.81]	[-0.94]
Price above VND 50k	1658***	355***	-576***	-535***	1245***	2172**	-186	-1970**
	[22.4]	[3.29]	[-7.48]	[-4.72]	[3.18]	[2.44]	[-0.47]	[-2.08]
Price between VND 10k and VND 50k	652***	25	-264***	-24	230***	38	-23	81
	[58.33]	[1.35]	[-20.51]	[-1.15]	[10.7]	[1.12]	[-0.96]	[1.48]
Price less than VND 10k	278***	-6	-127***	-17**	172***	32	-62***	-68**
	[67.31]	[-0.93]	[-26.66]	[-2.3]	[10.67]	[1.06]	[-3.84]	[-2.15]
	Effective spread in % of the price							
All stocks	3.34***	0.09	-1.54***	-0.22***	1.76***	0.52**	-0.46***	-0.47*
	[112.19]	[1.59]	[-43.99]	[-3.48]	[14.3]	[2.38]	[-3.6]	[-1.93]
Price above VND 50k	2.33***	0.29*	-0.99***	-0.53***	1.37***	2.88***	-0.17	-2.84***
	[20.5]	[1.81]	[-8.44]	[-3.16]	[4.28]	[2.74]	[-0.52]	[-2.61]
Price between VND 10k and VND 50k	3.1***	0.03	-1.39***	-0.02	1.37***	0.15	-0.43***	0.21
	[62.51]	[0.4]	[-24.71]	[-0.24]	[10.45]	[0.78]	[-3.1]	[0.92]
Price less than VND 10k	4.11***	0.07	-1.76***	-0.52***	2.75***	0.54	-0.87***	-1.07**
	[75.59]	[0.85]	[-28.49]	[-5.13]	[11.23]	[1.25]	[-3.52]	[-2.34]

The table presents the coefficient estimates and t-statistics in square brackets of the following regression:

*y*_*t*_ = *α* + *β*_1_*event*_*t*_ + *β*_2_*HSX*_*t*_ + *β*_3_*event*_*t*_ × *HSX*_*t*_ + *ϵ*_*t*_, where *event*_*t*_ equals 1 in days following the event and 0 otherwise, *HSX*_*t*_ equals 1 for stocks listed on the HSX and 0 otherwise, and the coefficient *β*_3_ illustrates the impact of minimum tick size change on the dependent variable *y*_*t*_ with the DID approach. The effective spreads are measured for each trade as twice the absolute difference between the trade price and the quote midpoint. The equal-weighted value on day *t* is computed as a simple average across trades for each stock and a simple average across stocks. The volume-weighted value on day *t* is computed as a share-weighted average across trades for each stock and a volume-weighted average across stocks. Before and after the tick size change, the period covers eight weeks before 12/9/2016 and eight weeks from 19/9/2016, respectively. Standard errors are adjusted for heteroskedasticity.

***, **, and * denote the statistical significance at the 1%, 5%, and 10% level, respectively.

When we study tick size change over different price ranges, stock prices above VND 50,000 display the most significant reduction in the effective spread at a 5% significance level, i.e., VND 535 and VND 1,970 (0.53% and 2.84%) on an equal- and volume-weighted basis, respectively. For stock prices less than VND 10,000, the decrease in the effective spread is statistically significant at a 5% significance level on both the equal- and volume-weighted approaches, i.e., VND 17 VND and VND 68 (0.52% and 1.07%). Meanwhile, the stock prices between VND 10,000 and VND 50,000 do not seem to benefit from the tick size reduction since the coefficient remains statistically insignificant in both weighting schemes.

The results so far do not consider different trade sizes, whereas Harris (1999) predicts that large traders might be disadvantaged by tick size reduction. Table 4 reports the average effective spreads for small (less than 1,000 shares), medium (1,000 to 9,999 shares), and large (10,000 or more shares) trades.

**Table 4:** The average effective bid-ask spreads by trade size.

	Equal-weighted				Volume-weighted			
	Constant	Event_t	HSX_t	Event_t* HSX_t	Constant	Event_t	HSX_t	Event_t* HSX_t
	**Panel A: All stocks**							
	Effective spread in VND							
Large trades	355***	126*	16	-176***	439***	362**	-182**	-206
	[8.9]	[1.94]	[0.38]	[-2.59]	[5.77]	[2.07]	[-2.35]	[-1.11]
Medium trades	409***	6	-62***	-53***	156***	2	39***	-91***
	[43.69]	[0.41]	[-5.69]	[-3.23]	[34.05]	[0.39]	[7.54]	[-13.91]
Small trades	631***	26	-195***	-63***	257***	31***	5	-143***
	[58.87]	[1.52]	[-15.49]	[-3.21]	[43.23]	[3.34]	[0.74]	[-14.35]
	Effective spread in % of the price							
Large trades	2.26***	0.56***	-0.74***	-0.87***	2.93***	1.07**	-1.4***	-0.75
	[22.86]	[3.19]	[-7.29]	[-4.77]	[9.55]	[2.11]	[-4.53]	[-1.4]
Medium trades	2.38***	0	-0.95***	-0.24***	1.13***	0.1***	-0.27***	-0.52***
	[59.58]	[-0.02]	[-21.9]	[-3.52]	[48.72]	[2.61]	[-9.95]	[-12.37]
Small trades	3.24***	0.13**	-1.53***	-0.27***	1.25***	0.16***	-0.45***	-0.52***
	[97.7]	[2.09]	[-40.14]	[-3.99]	[65.23]	[3.58]	[-20.04]	[-10.99]
	**Panel B: Large trades only**							
	Effective spread in VND							
Price above VND 50k	5200***	5286*	-3831**	-5272*	5169***	7145**	-3656*	-6330**
	[2.69]	[1.74]	[-1.98]	[-1.73]	[2.64]	[2.26]	[-1.86]	[-1.97]
Price between VND 10k and VND 50k	333***	99*	-55**	-109**	352***	107	-103**	119
	[12.53]	[1.85]	[-1.98]	[-1.98]	[7.25]	[1.25]	[-2.03]	[1.11]
Price less than VND 10k	169***	25	-45***	-75***	230***	70	-119***	-95*
	[19.87]	[1.58]	[-5.12]	[-4.59]	[7.56]	[1.23]	[-3.88]	[-1.65]
	Effective spread in % of the price							
Price above VND 50k	4.94***	7.49**	-3.2*	-7.81**	4.87***	10.01***	-3.18*	-9.43***
	[3.02]	[2.41]	[-1.95]	[-2.51]	[2.97]	[3]	[-1.93]	[-2.8]
Price between VND 10k and VND 50k	1.88***	0.42*	-0.64***	-0.47**	2.13***	0.47	-0.98***	0.27
	[14.96]	[1.82]	[-5]	[-1.99]	[7.4]	[1.05]	[-3.31]	[0.55]
Price less than VND 10k	2.73***	0.43*	-0.75***	-1.22***	3.88***	0.86	-1.95***	-1.19
	[19.67]	[1.87]	[-5.27]	[-5.1]	[7.69]	[1.05]	[-3.85]	[-1.42]

The table presents the coefficient estimates and t-statistics in square brackets of the following regression:

*y*_*t*_ = *α* + *β*_1_*event*_*t*_ + *β*_2_*HSX*_*t*_ + *β*_3_*event*_*t*_ × *HSX*_*t*_ + *ϵ*_*t*_, where *event*_*t*_ equals 1 in days following the event and 0 otherwise, *HSX*_*t*_ equals 1 for stocks listed on the HSX and 0 otherwise, and the coefficient *β*_3_ illustrates the impact of minimum tick size change on the dependent variable *y*_*t*_ with the DID approach. The effective spreads are measured for each trade as twice the absolute difference between the trade price and the quote midpoint. The equal-weighted value on day *t* is computed as a simple average across trades for each stock and a simple average across stocks. The volume-weighted value on day *t* is computed as a share-weighted average across trades for each stock and a volume-weighted average across stocks. Small trades are less than 1,000 shares, medium trades are from 1,000–9,999 shares, and large trades are 10,000 shares and over. Before and after the tick size change, the period covers eight weeks before 12/9/2016 and eight weeks from 19/9/2016, respectively. Standard errors are adjusted for heteroskedasticity.

***, **, and * denote the statistical significance at the 1%, 5%, and 10% level, respectively.

The effective spreads for medium and small trades decrease in both weighting schemes at a 1% significance level, i.e., VND 53 and VND 91 (0.24% and 0.52%) on the equal-weighted and volume-weighted basis, respectively, for the medium trades. The corresponding numbers for small trades are VND 63 and VND 143 (0.27% and 0.52%). The results for large trades are only statistically significant for the equal-weighted approach at a 1% level, i.e., VND 176 (0.87%). We repeat the analysis on different price ranges in Table 4 to better understand what drives the insignificant change in effective spreads of large trades. Large trades generally benefit from the tick size reduction when executed in the price range above VND 50,000, with the results statistically significant mostly at a 5% level in both weighting schemes, i.e., VND 5,272 and VND 6,330 (7.81% and 9.43%). The results are only statistically significant for the other price ranges at a 5% level on the equal-weighted basis, i.e., VND 109 (0.47%) or VND 75 (1.22%) with price ranges between VND 10,000 and 50,000 or less than VND 10,000. The change in percentage effective spread is less pronounced than that with a price range above VND 50,000. Overall, the results for large trades seemingly support the prediction in Harris (1999), whose reduction in percentage effective spread is less robust to different weighting schemes and price ranges.

### 4.4. Realized spreads

An alternative measure of trading cost is based on the realized spread, defined as twice the amount by which prices for customer buy orders exceed, or prices for customer sell orders fall short of, the estimated post-trade value of the asset. By construction, the metric attempts to measure the post-trade price reversal and represents a short-term measure of the potential profit or loss realized by traders who takes the other side of the order. Any decrease in the realized spread post-event period suggests the net gain to liquidity providers is close to zero. We follow Lee and Ready’s [28] algorithm to sign trades, and the midpoint of the quotes in effect 30 minutes after the trade, or the 14:30 midpoints for trades executed after 14:00, as a proxy for post-trade value.

Table 5 reports the average realized spread overall and for various trade size categories. On average, the reduction in a realized spread is statistically significant at a 5% level in the equal-weighted approach with a price range less than VND 10,000 or above VND 50,000, and it turns less statistically significant for the volume-weighted approach. In particular, the realized spread decreases by VND 40 (0.14%) overall, VND 462 (0.52%) for the stock price above VND 50,000, and VND 20 (0.49%) for a stock price less than VND 10,000. Meanwhile, the change in an effective spread in stock price between VND 10,000 and VND 50,000 occasionally carries a positive sign but is statistically insignificant in the volume-weighted approach.

**Table 5:** The average realized bid-ask spreads.

	Equal-weighted				Volume-weighted			
	Constant	Event_t	HSX_t	Event_t* HSX_t	Constant	Event_t	HSX_t	Event_t* HSX_t
	Realized spread in VND							
All stocks	374***	41**	-127***	-40**	135***	138**	-27	-20
	[36.99]	[2.5]	[-10.18]	[-2.1]	[4.85]	[2.39]	[-0.93]	[-0.28]
Price above VND 50k	805***	417***	-278***	-462***	653	2085**	-225	-1567*
	[11.27]	[3.26]	[-3.61]	[-3.45]	[1.64]	[2.37]	[-0.56]	[-1.68]
Price between VND 10k and VND 50k	411***	19	-185***	-3	110***	38	-17	98
	[30.19]	[0.94]	[-11.78]	[-0.12]	[4.06]	[0.96]	[-0.59]	[1.63]
Price less than VND 10k	163***	10	-77***	-20**	87***	33	-30*	-38
	[28.2]	[1.16]	[-12.22]	[-2.17]	[5.32]	[1.16]	[-1.76]	[-1.25]
	Realized spread in % of the price							
All stocks	2.03***	0.12*	-1.01***	-0.14*	0.92***	0.47**	-0.28*	-0.17
	[49.59]	[1.68]	[-21.47]	[-1.77]	[6.55]	[2.07]	[-1.86]	[-0.67]
Price above VND 50k	1.25***	0.44**	-0.58***	-0.52***	0.61*	2.84***	-0.11	-2.4**
	[11.18]	[2.46]	[-4.98]	[-2.8]	[1.82]	[2.74]	[-0.32]	[-2.23]
Price between VND 10k and VND 50k	1.98***	0	-0.99***	0.09	0.67***	0.15	-0.24	0.29
	[33.23]	[-0.05]	[-14.57]	[0.92]	[4.48]	[0.73]	[-1.51]	[1.18]
Price less than VND 10k	2.38***	0.22*	-1***	-0.49***	1.4***	0.53	-0.43	-0.56
	[31.93]	[1.95]	[-12.38]	[-3.88]	[5.48]	[1.21]	[-1.59]	[-1.17]
	Realized spread in VND							
Large trades	233***	123*	-53	-105	318***	354**	-180**	-156
	[5.57]	[1.93]	[-1.2]	[-1.55]	[3.85]	[2.12]	[-2.15]	[-0.87]
Medium trades	171***	15	-32***	-29*	39***	8	8	-32***
	[16.9]	[1]	[-2.63]	[-1.69]	[7.26]	[0.93]	[1.05]	[-3.03]
Small trades	381***	42**	-136***	-48**	75***	36***	1	-86***
	[34.91]	[2.41]	[-10.27]	[-2.37]	[10.4]	[3.5]	[0.08]	[-7.23]
	Realized spread in % of the price							
Large trades	1.21***	0.5**	-0.49***	-0.54***	1.97***	1.05**	-1.16***	-0.47
	[9.29]	[2.5]	[-3.62]	[-2.62]	[5.78]	[1.99]	[-3.32]	[-0.85]
Medium trades	1.13***	0	-0.48***	-0.07	0.34***	0.06	-0.04	-0.25***
	[25.16]	[-0.04]	[-9.62]	[-0.9]	[10.69]	[1.45]	[-0.96]	[-4.74]
Small trades	2.02***	0.15**	-1***	-0.23***	0.45***	0.11***	-0.14***	-0.31***
	[45.48]	[2.09]	[-20.14]	[-2.84]	[17.95]	[3.14]	[-4.21]	[-7.47]

The table presents the coefficient estimates and t-statistics in square brackets of the following regression:

*y*_*t*_ = *α* + *β*_1_*event*_*t*_ + *β*_2_*HSX*_*t*_ + *β*_3_*event*_*t*_ × *HSX*_*t*_ + *ϵ*_*t*_, where *event*_*t*_ equals 1 in days following the event and 0 otherwise, *HSX*_*t*_ equals 1 for stocks listed on the HSX and 0 otherwise, and the coefficient *β*_3_ illustrates the impact of minimum tick size change on the dependent variable *y*_*t*_ with the DID approach. The realized spreads are measured for each trade as the buy-sell indicator variable times twice the difference between the price and the quote midpoint 30 minutes after the trade. The equal-weighted value on day *t* is computed as a simple average across trades for each stock and a simple average across stocks. The volume-weighted value on day *t* is computed as a share-weighted average across trades for each stock and a volume-weighted average across stocks. Small trades are less than 1,000 shares, medium trades are from 1,000–9,999 shares, and large trades are 10,000 shares and over. Before and after the tick size change, the period covers eight weeks before 12/9/2016 and eight weeks from 19/9/2016, respectively. Standard errors are adjusted for heteroskedasticity.

***, **, and * denote the statistical significance at the 1%, 5%, and 10% level, respectively.

We also report the results for different trade sizes in Table 5 to document that large trades seemingly do not benefit from reducing tick size. On average, the small and medium trades enjoy a reduction in realized spreads by VND 48 (0.23%) and VND 239 under the equal-weighted approach and VND 86 (0.31%) and VND 32 (0.25%) under the volume-weighted approach. The results are more statistically significant at a 1% level for small trades. Meanwhile, the corresponding value decreases by 0.54% at a 1% significance level in the equal-weighted approach for large trades. The reduction in a realized spread for small and medium trades implies that liquidity is still available for these trade sizes even though the net gain to liquidity providers is near zero.

The results until this point suggest that the realized spreads of either large trades or price ranges between VND 10,000 and VND 50,000 are not substantially reduced as expected. Therefore, we further study the impact of tick size change on trade size with a price range between VND 10,000 and VND 50,000 in Table 6. The results are statistically insignificant and even carry a positive sign in some cases, except that the realized spread reduces by VND 30 (0.1%) for small trades in the volume-weighted method. As a comparison, we report the results on the trade size with a price range above VND 50,000 in the second panel of Table 6 to report that the realized spread decreases by VND 4,450 (7.03%) or VND 5,491 (8.5%) for large trades; VND 322 (0.37%) or VND 541 (0.75%) for the medium trades; VND 459 (0.48%) or VND 356 (0.37%) for the small trades, across the equal- and volume-weighted approach. In addition, the results are statistically significant at a 5% level in most cases.

**Table 6:** Average realized bid-ask spreads by trade size for the specific price range.

	Equal-weighted				Volume-weighted			
	Constant	Event_t	HSX_t	Event_t* HSX_t	Constant	Event_t	HSX_t	Event_t* HSX_t
	**Panel A: Price between 10,000 and 50,000 VND**							
	Realized spread in VND							
Large trades	195***	86	-61*	-68	225***	123	-95	111
	[5.99]	[1.4]	[-1.79]	[-1.08]	[3.57]	[1.24]	[-1.46]	[0.93]
Medium trades	203***	0	-80***	0	37***	4	-5	-12
	[14.87]	[-0.02]	[-5.4]	[-0.01]	[5.54]	[0.33]	[-0.58]	[-0.84]
Small trades	410***	27	-192***	-12	75***	19**	-37***	-30***
	[30.93]	[1.25]	[-12.45]	[-0.48]	[12.66]	[2.04]	[-5.62]	[-3.02]
	Realized spread in % of the price							
Large trades	1.07***	0.32	-0.48***	-0.24	1.37***	0.5	-0.76**	0.28
	[6.71]	[1.18]	[-2.94]	[-0.88]	[4.18]	[1.04]	[-2.26]	[0.53]
Medium trades	1.02***	-0.09	-0.46***	0.11	0.22***	0.01	-0.08**	-0.04
	[16.1]	[-0.99]	[-6.69]	[1.2]	[7.34]	[0.19]	[-2.25]	[-0.7]
Small trades	1.95***	0.04	-1***	0.02	0.37***	0.05	-0.21***	-0.1***
	[34.55]	[0.49]	[-15.38]	[0.23]	[14.94]	[1.48]	[-7.72]	[-2.67]
	**Panel B: Price above 50,000 VND**							
	Realized spread in VND							
Large trades	5217**	4729	-4536**	-4450	5129**	6676**	-4302**	-5491*
	[2.57]	[1.53]	[-2.23]	[-1.44]	[2.52]	[2.13]	[-2.11]	[-1.72]
Medium trades	198***	267**	102	-322**	23	476***	102**	-541***
	[2.89]	[2.13]	[1.41]	[-2.48]	[0.59]	[3.05]	[2.35]	[-3.42]
Small trades	833***	395***	-302***	-459***	127***	201***	65*	-356***
	[11.11]	[3.02]	[-3.75]	[-3.35]	[4]	[4.08]	[1.76]	[-6.57]
	Realized spread in % of the price							
Large trades	4.69***	7.05**	-3.77**	-7.03**	4.58***	9.55***	-3.64**	-8.5**
	[2.65]	[2.19]	[-2.13]	[-2.18]	[2.63]	[2.84]	[-2.08]	[-2.5]
Medium trades	0.33***	0.31*	0.06	-0.37**	0.05	0.66***	0.11*	-0.75***
	[4.58]	[1.86]	[0.76]	[-2.2]	[1.01]	[2.61]	[1.91]	[-2.92]
Small trades	1.3***	0.38**	-0.63***	-0.48**	0.18***	0.2***	0.04	-0.37***
	[10.77]	[2.05]	[-5.03]	[-2.51]	[4.44]	[3.44]	[0.99]	[-6.04]

The table presents the coefficient estimates and t-statistics in square brackets of the following regression:

*y*_*t*_ = *α* + *β*_1_*event*_*t*_ + *β*_2_*HSX*_*t*_ + *β*_3_*event*_*t*_ × *HSX*_*t*_ + *ϵ*_*t*_, where *event*s equal 1 in days following the event and 0 otherwise, *HSX*_*t*_ equals 1 for stocks listed on the HSX and 0 otherwise, and the coefficient *β*_3_ illustrates the impact of minimum tick size change on the dependent variable *y*_*t*_ with the DID approach. The realized spreads are measured for each trade as the buy-sell indicator variable times twice the difference between the price and the quote midpoint 30 minutes after the trade. The equal-weighted value on day *t* is computed as a simple average across trades for each stock and a simple average across stocks. The volume-weighted value on day *t* is computed as a share-weighted average across trades for each stock and a volume-weighted average across stocks. Small trades are less than 1,000 shares, medium trades are from 1,000–9,999 shares, and large trades are 10,000 shares and over. Before and after the tick size change, the period covers eight weeks before 12/9/2016 and eight weeks from 19/9/2016, respectively. Standard errors are adjusted for heteroskedasticity.

***, **, and * denote the statistical significance at the 1%, 5%, and 10% level, respectively.

Overall, we find consistent results with the effective spread in that large trades do not benefit from the decrease in tick size, especially those executed at prices between VND 10,000 and VND 50,000. However, regardless of trade sizes, the realized spread associated with a price less than VND 10,000 or VND 50,000 displays a significant reduction following the tick size step of VND 10 or VND 100.

### 4.5. Spreads by location of trade price relative to the quote

In the US market, anecdotal evidence indicates large traders using limit orders increasingly complain of being “pennied” by liquidity providers who improve the price by the minimum increment. In the Vietnamese context, we investigate this issue by studying the quoted, effective, and realized spreads for three categories of trades: execution outside the quote, at the quote, and inside the quote in Table 7. Consistent with the previous results, the quoted spreads substantially decrease for each trade category post-event period, i.e., by VND 243 (0.66%) and VND 330 (2.09%) for trades inside quotes on the equally- and volume-weighted basis, respectively. The corresponding numbers are VND 68 (0.23%) and VND 97 (0.88%) for trades at quotes and VND 109 (0.33%), and VND 109 (0.41%) for trades outside quotes. Moreover, the results are mainly statistically significant at a 1% level.

**Table 7:** Average quoted, effective, and realized bid-ask spreads by location of trade price relative to the quote.

	Equal-weighted				Volume-weighted			
	Constant	Event_t	HSX_t	Event_t* HSX_t	Constant	Event_t	HSX_t	Event_t* HSX_t
	Quoted spread in VND							
Trades inside quotes	915***	41	-323***	-243***	686***	-25	69	-330*
	[19.48]	[0.61]	[-5.49]	[-3.07]	[14.33]	[-0.37]	[0.48]	[-1.84]
Trades at quotes	556***	31**	-150***	-68***	141***	5	16***	-97***
	[64.82]	[2.15]	[-14.06]	[-3.91]	[52.71]	[1.22]	[4.39]	[-19.66]
Trades outside quotes	512***	11	-146***	-109***	330***	-18	-100**	-109**
	[32.86]	[0.44]	[-8.76]	[-4.18]	[8.5]	[-0.33]	[-2.47]	[-1.97]
	Quoted spread in % of the price							
Trades inside quotes	3.66***	0.04	-1.8***	-0.66***	3.5***	0.67	-0.82*	-2.09***
	[29.17]	[0.21]	[-10.97]	[-2.98]	[13.13]	[1.29]	[-1.68]	[-3.07]
Trades at quotes	3***	0.07	-1.37***	-0.23***	1.08***	0.21***	-0.06**	-0.88***
	[97.22]	[1.31]	[-37.95]	[-3.72]	[50.28]	[2.99]	[-2.14]	[-12.26]
Trades outside quotes	2.23***	0.08	-0.96***	-0.33***	1.92***	-0.14	-0.86***	-0.41
	[49.47]	[1]	[-19.88]	[-4.15]	[9.85]	[-0.57]	[-4.24]	[-1.61]
	Effective spread in VND							
Trades inside quotes	480***	-4	-277***	4	331***	-58	-19	-115
	[11.69]	[-0.08]	[-5.53]	[0.06]	[8.21]	[-1.09]	[-0.16]	[-0.9]
Trades at quotes	562***	31**	-153***	-66***	143***	4	14***	-94***
	[64.76]	[2.11]	[-14.17]	[-3.81]	[44.85]	[0.83]	[3.47]	[-17.61]
Trades outside quotes	1073***	49	-82***	-453***	1488***	997*	-307	-1225**
	[40.37]	[1.23]	[-2.69]	[-10.31]	[5.8]	[1.75]	[-1.15]	[-2.09]
	Effective spread in % of the price							
Trades inside quotes	1.65***	-0.02	-1***	0.02	1.42***	0.26	-0.33	-0.93*
	[14.74]	[-0.1]	[-7.1]	[0.1]	[5.46]	[0.66]	[-0.76]	[-1.72]
Trades at quotes	3.02***	0.07	-1.38***	-0.23***	1.09***	0.21***	-0.06**	-0.88***
	[97.74]	[1.28]	[-38.13]	[-3.64]	[48.49]	[2.86]	[-2.27]	[-11.58]
Trades outside quotes	5.27***	0.29**	-1.98***	-1.37***	8.55***	1.5	-3.79***	-2.13**
	[58.09]	[2.05]	[-20.3]	[-9.07]	[12.58]	[1.49]	[-5.32]	[-2]
	Realized spread in VND							
Trades inside quotes	383***	12	-143**	-43	228***	24	-86	11
	[7.1]	[0.17]	[-2.11]	[-0.51]	[4.44]	[0.33]	[-1.02]	[0.07]
Trades at quotes	310***	46***	-100***	-45**	23***	8	7	-35**
	[34.45]	[2.98]	[-8.64]	[-2.4]	[2.69]	[0.63]	[0.66]	[-2.48]
Trades outside quotes	717***	43	-73**	-327***	1257***	974*	-291	-1118**
	[25.87]	[1.16]	[-2.25]	[-7.62]	[4.79]	[1.81]	[-1.06]	[-2.01]
	Realized spread in % of the price							
Trades inside quotes	1.29***	0.04	-0.58**	-0.14	1.01***	0.27	-0.66	-0.25
	[6.73]	[0.17]	[-2.49]	[-0.52]	[3.06]	[0.68]	[-1.59]	[-0.49]
Trades at quotes	1.76***	0.1	-0.86***	-0.13*	0.28***	0.17	0.07	-0.5***
	[42.54]	[1.42]	[-18.33]	[-1.74]	[6.38]	[1.41]	[1.11]	[-3.71]
Trades outside quotes	3.64***	0.26*	-1.53***	-0.99***	6.97***	1.5	-3.06***	-1.91*
	[36.66]	[1.84]	[-14.25]	[-6.44]	[10]	[1.55]	[-4.15]	[-1.86]

The table presents the coefficient estimates and t-statistics in square brackets of the following regression:

*y*_*t*_ = *α* + *β*_1_*event*_*t*_ + *β*_2_*HSX*_*t*_ + *β*_3_*event*_*t*_ × *HSX*_*t*_ + *ϵ*_*t*_, where *event*_*t*_ equals 1 in days following the event and 0 otherwise, *HSX*_*t*_ equals 1 for stocks listed on the HSX and 0 otherwise, and the coefficient *β*_3_ illustrates the impact of minimum tick size change on the dependent variable *y*_*t*_ with the DID approach. The quoted spread is recorded at the time of each trade. The effective spreads are measured for each trade as twice the absolute difference between the trade price and the quote midpoint. The realized spreads are measured for each trade as the buy-sell indicator variable times twice the difference between the price and the quote midpoint 30 minutes after the trade. The equal-weighted value on day *t* is computed as a simple average across trades for each stock and a simple average across stocks. The volume-weighted value on day *t* is computed as a share-weighted average across trades for each stock and a volume-weighted average across stocks. Before and after the tick size change, the period covers eight weeks before 12/9/2016 and eight weeks from 19/9/2016, respectively. Standard errors are adjusted for heteroskedasticity.

***, **, and * denote the statistical significance at the 1%, 5%, and 10% level, respectively.

Concerning the effective spread, the results are statistically significant at a 5% level for trades at and outside the quotes. The effective spread decreases by VND 66 (0.23%) and VND 94 (0.88%) for trades at quotes on the equally- and volume-weighted basis, respectively. The corresponding numbers are VND 453 (1.37%) and VND 1,225 (2.13%) for trades outside quotes. Similar results are reported for the realized spread, with statistically significant results at a 5% level in many cases for trades at and outside the quotes. The realized spread decreases by VND 45 (0.13%) and VND 35 (0.5%) for trades at quotes on the equally- and volume-weighted basis, respectively. The corresponding numbers are VND 327 (0.99%) and VND 1,118 (1.91%) for trades outside quotes.

The results so far have shown that trades within the quotes contain less information in that they move subsequent midpoints less than those executed at the quotes. It is also consistent with Bessembinder’s [19] evidence that discretionary liquidity providers do not step ahead of the quote to offer price improvement to market orders but "cream skims" orders originating from less informed traders. In addition, trades completed outside the quotes still pay substantially more significant execution costs measured by either effective or realized spreads after the tick size change. In summary, we document a similar finding in [19] that trades within the quotes do not enjoy a reduction in realized spread after the tick size change, and trades outside the quotes witness the largest reduction in trading costs proxied by effective and realized spreads.

### 4.6. Quotation Size and market volatility

Harris [10] predicts that a smaller tick size can prevent liquidity supply following a reduced tick size. The liquidity supply increases trade execution costs for large traders and price volatility. Table 8 reports that the quotation size decreases overall and across different price ranges, with the most substantial effect for the quotes less than VND 10,000, followed by prices above VND 50,000 and between VND 10,000 and VND 50,000. The results are statistically significant at a 1% level. On average, the quoted size decreased by 25,491 shares. The results are consistent with [19] and the references therein. We next investigate if price volatility indeed increases. We compute the stock volatility as the standard deviation of daily open-to-close midpoint quote returns. The second panel in Table 8 shows that overall return volatility remains unchanged rather than increased after the tick size change, while the return volatility of prices above VND 50,000 reduces by 0.57%, statistically significant at the 1% level. In other words, there is no indication of a reduction in liquidity after the minimum tick size change.

**Table 8:** The average quote sizes and returns volatility.

	Constant	Event_t	HSX_t	event_t* HSX_t
	NBBO quote size			
All stocks	6459***	587***	25765***	-25491***
	[69.53]	[2.58]	[37.44]	[-33.8]
Price above VND 50k	1128***	-197***	8397***	-7515***
	[40.94]	[-4.66]	[21.55]	[-19.08]
Price between VND 10k and VND 50k	5742***	545	8094***	-6322***
	[56.14]	[1.59]	[26.44]	[-11.23]
Price less than VND 10k	9514***	517*	89969***	-90477***
	[48.99]	[1.7]	[37.06]	[-36.84]
	Volatility			
All stocks	2.18***	-0.06	-0.52***	-0.1
	[27.55]	[-0.51]	[-4.81]	[-0.65]
Price above VND 50k	1.75***	0.18	0.03	-0.57***
	[14.75]	[1.04]	[0.21]	[-2.76]
Price between VND 10k and VND 50k	1.97***	0.1	-0.38***	-0.19
	[23.89]	[0.81]	[-3.36]	[-1.18]
Price less than VND 10k	2.44***	-0.29	-0.62**	0.12
	[13.76]	[-1.13]	[-2.33]	[0.32]

The table presents the coefficient estimates and t-statistics in square brackets of the following regression:

*y*_*t*_ = *α* + *β*_1_*event*_*t*_ + *β*_2_*HSX*_*t*_ + *β*_3_*event*_*t*_ × *HSX*_*t*_ + *ϵ*_*t*_, where *event*_*t*_ equals 1 in days following the event and 0 otherwise, *HSX*_*t*_ equals 1 for stocks listed on the HSX and 0 otherwise, and the coefficient *β*_3_ illustrates the impact of minimum tick size change on the dependent variable *y*_*t*_ with the DID approach. The size of each quote or (bid quote + ask quote)/2 is recorded, and the average quote size for each stock is obtained as the time-weighted mean of the individual quote sizes. The simple average quote size across stocks is calculated for each day *t*. The stock volatility is calculated as the standard deviation of daily open-to-close midpoint quote returns over the pre-and post-event period separately. To run the DID approach, we replace the day index *t* with stock index *i* in the abovementioned regression. Before and after the tick size change, the period covers eight weeks before 12/9/2016 and eight weeks from 19/9/2016, respectively. Standard errors are adjusted for heteroskedasticity.

***, **, and * denote the statistical significance at the 1%, 5%, and 10% level, respectively.

### 4.7. Robustness check

We have established the results that the trading cost reduces following the change of minimum tick size, but the results are less pronounced for stock prices between VND 10,000 and VND 50,000 and large trades. We continue to test the robustness of our results in Tables 1 to 8 by extending the sample period to 60 trading days over 16/6/2016-9/12/2016 before and after the policy change. We note that the results remain both quantitatively and qualitatively similar. The results are available upon request. We next include the cross-sectional determinants of liquidity and report the main coefficients of interest in the S1 Appendix. We note that such determinants are more relevant to the context of cross-sectional regression instead of time-series regression. Overall, the coefficient sign remains unchanged with statistical significance, mainly at the 5% level for the quoted spread. Moving to the effective and realized spread, the coefficient sign is still negative but less statistically significant. More interestingly, we find that both the effective and realized spreads with a price range between VND 10,000 and VND 50,000 increase at the weak significance level of 10%, and the coefficient sign changes statistically insignificantly positive. With respect to the large trades, we document similar results of the realized spreads with stock prices between VND 10,000 and VND 50,000 and a reduction in statistical significance for the results of realized spreads and stock prices above VND 50,000.

## 5. Conclusions and policy implications

The establishment of the stock markets and their contributions to sustainable economic growth and development in emerging markets have consistently attracted significant attention from policymakers, academics, and practitioners. In achieving this vital contribution to the stock markets, market quality is a significant factor to be considered. For an efficient operation of the markets to ensure market quality, the minimum tick size is a crucial factor for any consideration. Vietnam introduced and implemented the change in the minimum price change, the minimum tick size, in 2016 to support the stock market’s liquidity. However, the effect of this regulation is unknown. While the effect has been widely examined in developed markets, the effect of this important policy has largely been ignored in emerging markets such as Vietnam. This study examines the relationship between the minimum tick size and market quality in Vietnam’s stock markets.

The study uses both equally-weighted and volume-weighted approaches. The trade and quote intraday data of all stocks listed on the Ho Chi Minh Stock Exchange (HSX) are collected from Thomson Reuters. We consider the period from 1 January 2016 to 11 November 2016 using the trading hours range from 9:15 to 11:30 and 13:00 to 14:30. It is noted that Vietnam introduced the tick size change on 12 September 2016. Considering the effect of this decision on market quality, the four-week periods before the event, from 18/7/2016 to 11/9/2016, and after this event, from 19/9/2016 to 11/11/2016, are considered. One week between these two periods, from 12/9/2016 to 18/9/2016, is left out of the sample because we consider it essential to give the market some time to adapt to the new tick size policy.

Key findings from this paper can be summarized as follows. *First*, introducing tick size change reduces trading costs, as measured by the quoted spreads. *Second*, when the tick size changes over different price ranges are considered, stock prices less than VND10,000 display the greatest reduction in the effective spread. Besides, the stock prices between VND10,000 and VND50,000 do not benefit from the tick size reduction. Moreover, when different trade sizes are taken into consideration, we find that (i) the effective spreads for large trades exceed those for small trades following the tick size change; and (ii) large trades benefit from the tick size reduction when they are only executed in the price range less than VND 10,000. *Third*, large trades do not benefit from the decrease in tick size, especially those executed at prices above VND10,000. Finally, regardless of trade sizes, the realized spread associated with a price less than VND10,000 displays a significant reduction following the tick size step of VND10.

We note that the findings from this paper for Vietnam’s stock market are similar to the US market. There is no indication of a reduction in liquidity following a reduction in tick size. The implication for policy based on these trading is that the trading costs do not reduce for large trades and stock prices above VND 10,000. We consider this finding to be explained by the observation that the tick size increment is only VND 50 and VND 100 for stock prices between VND 10,000 and VND 50,000 and above VND 50,000. Meanwhile, trading costs reduce in the price range of less than VND 10,000, where the tick size increment is smallest at VND 10, irrespective of the trade size. Findings from this paper indicate that trading costs can be potentially reduced with the smallest tick size of VND 10 applied for stock prices above VND 10,000, where large trades can benefit the most. These findings imply that introducing a change in tick size in Vietnam in 2016 is desirable for improving market quality. However, the differentiation of these changes in different ranges of stock prices listed in Vietnam’s stock market is not necessarily effective for improving market quality.

## Supporting information

S1 Appendix The average quoted, effective and realized bid-ask spreads with controls. (DOCX)
