# Supplementary material for: Minimum tick size, market quality and costs of trade execution in Vietnam
Source: PLoS One. 2023 May 18;18(5):e0285821. doi: 10.1371/journal.pone.0285821 (PMC10194971; doi:10.1371/journal.pone.0285821)
Supplement: S1 Appendix — (DOCX) [file pone.0285821.s001.docx]

**Appendix: The average quoted, effective and realized bid-ask spreads with controls**

|  | Equal-weighted | | | |  | Volume-weighted | | | |
| --- | --- | --- | --- | --- | --- | --- | --- | --- | --- |
|  | Constant | Event_t | HSX_t | Event_t*  HSX_t |  | Constant | Event_t | HSX_t | Event_t*  HSX_t |
|  | Quoted spread in VND | | | | | | | | |
| All stocks | 3633 | 32** | 824** | -65*** |  | 148 | -3 | 132 | -80*** |
|  | [1.63] | [1.99] | [2.12] | [-3.85] |  | [0.13] | [-0.39] | [0.74] | [-10.02] |
| Price above VND 50k | 5578 | 286* | -639 | -440* |  | 13581 | 340** | 873 | -794*** |
|  | [0.44] | [1.65] | [-0.5] | [-1.67] |  | [1.01] | [2.26] | [1.54] | [-4.94] |
| Price between VND 10k and VND 50k | -134 | 12 | 158 | 32 |  | 2044 | 1 | 85 | -35*** |
|  | [-0.06] | [0.77] | [1.35] | [1.43] |  | [1.41] | [0.1] | [1.15] | [-2.63] |
| Price less than VND 10k | -1426** | 0 | -23 | -19** |  | -562 | 3 | 43 | -77*** |
|  | [-2.32] | [0.01] | [-0.71] | [-1.96] |  | [-0.75] | [0.31] | [1.08] | [-7.85] |
|  | Quoted spread in % of the price | | | | | | | | |
| All stocks | 35.44*** | 0.17*** | 3.58** | -0.25*** |  | 21.84** | 0.14** | 3.48** | -0.75*** |
|  | [4.21] | [2.66] | [2.43] | [-3.79] |  | [2.37] | [2.34] | [2.47] | [-11.88] |
| Price above VND 50k | 33.5* | 0.45* | -1.23 | -0.56 |  | 26.96 | 0.45 | 0.71 | -0.93*** |
|  | [1.91] | [1.92] | [-0.7] | [-1.51] |  | [1.04] | [1.61] | [1.05] | [-3.66] |
| Price between VND 10k and VND 50k | 21.92** | 0.09 | 0.58 | 0.11 |  | 15.05* | -0.01 | 0.5* | -0.11** |
|  | [2.13] | [1.45] | [1.16] | [1.18] |  | [1.91] | [-0.23] | [1.77] | [-1.99] |
| Price less than VND 10k | -0.44 | 0.04 | -0.71 | -0.59*** |  | 14.19 | 0.14 | 0.54 | -1.49*** |
|  | [-0.05] | [0.44] | [-1.44] | [-4.22] |  | [0.88] | [0.76] | [0.62] | [-7.5] |
|  | Effective spread in VND | | | | | | | | |
| All stocks | 3401 | 35* | 1183*** | -64*** |  | -2116 | 124** | 678 | 61 |
|  | [1.39] | [1.92] | [2.65] | [-3.46] |  | [-0.24] | [2.04] | [0.52] | [0.92] |
| Price above VND 50k | 5615 | 369* | -292 | -538* |  | -7549 | 1961*** | -3015 | -219 |
|  | [0.41] | [1.92] | [-0.21] | [-1.9] |  | [-0.14] | [3.03] | [-0.54] | [-0.19] |
| Price between VND 10k and VND 50k | -2667 | -4 | 221* | 62** |  | -6390 | -2 | -78 | 177* |
|  | [-1.02] | [-0.2] | [1.79] | [2.39] |  | [-0.81] | [-0.04] | [-0.26] | [1.76] |
| Price less than VND 10k | -1319* | 4 | -29 | -11 |  | -903 | -30 | -200* | 22 |
|  | [-1.94] | [0.57] | [-0.82] | [-1] |  | [-0.43] | [-1.13] | [-1.68] | [0.7] |
|  | Effective spread in % of the price | | | | | | | | |
| All stocks | 38.8*** | 0.21*** | 5.02*** | -0.26*** |  | 44.87 | 0.45** | 12.46** | -0.07 |
|  | [4.28] | [3.04] | [3.06] | [-3.8] |  | [1.54] | [2.1] | [2.31] | [-0.32] |
| Price above VND 50k | 35.75* | 0.62** | -0.5 | -0.76* |  | 18.85 | 2.77*** | -5.5 | -0.7 |
|  | [1.8] | [2.25] | [-0.24] | [-1.8] |  | [0.26] | [3.89] | [-0.86] | [-0.56] |
| Price between VND 10k and VND 50k | 16.32 | 0.05 | 0.84 | 0.22* |  | 7.23 | 0.14 | 0.37 | 0.53* |
|  | [1.36] | [0.58] | [1.47] | [1.93] |  | [0.15] | [0.76] | [0.27] | [1.77] |
| Price less than VND 10k | 6.05 | 0.09 | -0.83 | -0.48*** |  | 26.62 | -0.5 | -3.71* | 0.22 |
|  | [0.67] | [0.88] | [-1.61] | [-3.17] |  | [0.81] | [-1.32] | [-1.74] | [0.44] |
|  | Realized spread in VND | | | | | | | | |
| All stocks | 4753* | 48** | 755* | -43** |  | 5266 | 147** | 1311 | 88 |
|  | [1.79] | [2.56] | [1.84] | [-2.17] |  | [0.5] | [2.52] | [1] | [1.32] |
| Price above VND 50k | -5959 | 219 | -3155 | 67 |  | -19015 | 1653** | -6145 | 688 |
|  | [-0.33] | [0.98] | [-1.48] | [0.18] |  | [-0.35] | [2.5] | [-1.09] | [0.59] |
| Price between VND 10k and VND 50k | 1155 | 10 | 347** | 62* |  | -3213 | 3 | 131 | 208* |
|  | [0.37] | [0.39] | [2.37] | [1.71] |  | [-0.34] | [0.06] | [0.34] | [1.81] |
| Price less than VND 10k | -1589* | 18** | -41 | -26* |  | 2481 | -41 | -309** | 42 |
|  | [-1.81] | [1.97] | [-0.77] | [-1.87] |  | [1.3] | [-1.62] | [-2.5] | [1.41] |
|  | Realized spread in % of the price | | | | | | | | |
| All stocks | 26.53*** | 0.2** | 3.35** | -0.17** |  | 62.34 | 0.49** | 14.17** | 0.15 |
|  | [2.68] | [2.55] | [1.98] | [-2.23] |  | [1.57] | [2.19] | [2.53] | [0.65] |
| Price above VND 50k | 4.63 | 0.37 | -3.46 | -0.05 |  | 4.9 | 2.49*** | -8.6 | 0.27 |
|  | [0.2] | [1.17] | [-1.17] | [-0.1] |  | [0.07] | [3.39] | [-1.31] | [0.21] |
| Price between VND 10k and VND 50k | 21.02 | 0.03 | 1.18* | 0.28* |  | 15.47 | 0.17 | 0.59 | 0.55 |
|  | [1.5] | [0.31] | [1.76] | [1.94] |  | [0.29] | [0.79] | [0.36] | [1.58] |
| Price less than VND 10k | -9.11 | 0.28** | -0.9 | -0.62*** |  | 79.4* | -0.73** | -6.22*** | 0.42 |
|  | [-0.77] | [2.19] | [-1.23] | [-3.39] |  | [1.86] | [-1.99] | [-2.64] | [0.71] |
|  | Realized spread in VND and price between VND 10k and VND 50k | | | | | | | | |
| Large trades | -6454 | 12 | 366 | 47 |  | 443 | 98 | 213 | 251 |
|  | [-0.81] | [0.17] | [0.88] | [0.48] |  | [0.02] | [0.87] | [0.28] | [1.25] |
| Medium trades | 4588 | -4 | 330*** | 40 |  | 3840*** | 1 | 283*** | 14 |
|  | [1.63] | [-0.18] | [2.77] | [1.17] |  | [2.64] | [0.12] | [2.87] | [0.61] |
| Small trades | 612 | 23 | 225 | 34 |  | 1511 | 17 | 39 | -33** |
|  | [0.18] | [0.9] | [1.44] | [0.89] |  | [1.27] | [1.63] | [0.56] | [-2.24] |
|  | Realized spread in % of price and price between VND 10k and VND 50k | | | | | | | | |
| Large trades | -43.45 | -0.04 | 1.07 | 0.29 |  | 45.2 | 0.68 | 0.87 | 0.57 |
|  | [-1.02] | [-0.1] | [0.64] | [0.66] |  | [0.4] | [1.29] | [0.26] | [0.79] |
| Medium trades | 16.14 | -0.11 | 1.08* | 0.27* |  | 16*** | 0.01 | 0.98** | 0.05 |
|  | [1.3] | [-1.01] | [1.91] | [1.75] |  | [2.61] | [0.13] | [2.53] | [0.57] |
| Small trades | 21.49 | 0.12 | 0.69 | 0.1 |  | 9.04* | 0.07* | -0.01 | -0.16*** |
|  | [1.46] | [1.29] | [1.04] | [0.72] |  | [1.95] | [1.67] | [-0.03] | [-2.66] |
|  | Realized spread in VND and price above VND 50k | | | | | | | | |
|  | Realized spread in VND | | | | | | | | |
| Large trades | 199749 | 3868 | -12183 | -1989 |  | 245848 | 6699 | 1584 | -4363 |
|  | [0.79] | [0.78] | [-0.52] | [-0.32] |  | [1.11] | [1.63] | [0.07] | [-0.82] |
| Medium trades | -12682 | 21 | -3330 | 268 |  | -27273 | 260 | 625 | -400 |
|  | [-0.92] | [0.11] | [-1.56] | [0.73] |  | [-1.6] | [1.1] | [0.19] | [-0.77] |
| Small trades | -2879 | 257 | -2715 | -24 |  | 3670 | 191*** | -182 | -306*** |
|  | [-0.16] | [1.1] | [-1.24] | [-0.06] |  | [0.65] | [3.1] | [-0.25] | [-2.89] |
|  | Realized spread in % of price and price above VND 50k | | | | | | | | |
| Large trades | 324.03 | 7.39 | -13.43 | -5.42 |  | 369.92 | 10.33*** | -0.69 | -7.65 |
|  | [1.09] | [1.64] | [-0.65] | [-0.94] |  | [1.39] | [2.98] | [-0.04] | [-1.61] |
| Medium trades | -12.11 | 0.1 | -2.31 | 0.09 |  | -20.85 | 0.54 | 4.42 | -1.08 |
|  | [-0.66] | [0.4] | [-0.69] | [0.17] |  | [-0.89] | [1.5] | [0.79] | [-1.24] |
| Small trades | 4.4 | 0.34 | -3.45 | -0.03 |  | 1.3 | 0.18*** | -0.45 | -0.27** |
|  | [0.19] | [1.06] | [-1.12] | [-0.05] |  | [0.21] | [2.63] | [-0.6] | [-2.33] |

The table presents the coefficient estimates and t-statistics in square brackets of the following regression:

$y_{t}= \alpha+\beta_{1}event_{t}+\beta_{2}HSX_{t}+\beta_{3}event_{t}\times HSX_{t}+controls_{t}+\epsilon_{t}$, where $event_{t}$ equals 1 in days following the event and 0 otherwise, ${HSX}_{t}$ equals 1 for stocks listed on the HSX and 0 otherwise, and the coefficient $\beta_{3}$ illustrates the impact of minimum tick size change on the dependent variable $y_{t}$ with the DID approach. We also control for the cross-sectional determinants of liquidity mentioned in the methodology section. The quoted spread is recorded at the time of each trade. The effective spreads are measured for each trade as twice the absolute difference between the trade price and the quote midpoint. The realized spreads are measured for each trade as the buy-sell indicator variable times twice the difference between the price and the quote midpoint 30 minutes after the trade. The equal-weighted value on day $t$ is computed as a simple average across trades for each stock and a simple average across stocks. The volume-weighted value on day $t$ is computed as a share-weighted average across trades for each stock and a volume-weighted average across stocks. Small trades are less than 1,000 shares, medium trades are from 1,000-9,999 shares, and large trades are 10,000 shares and over. Before and after the tick size change, the period covers eight weeks before 12/9/2016 and eight weeks from 19/9/2016, respectively. Standard errors are adjusted for heteroskedasticity. ***, **, and * denote the statistical significance at the 1%, 5%, and 10% level, respectively.
